# Developmental environment shapes honeybee worker response to virus infection

**DOI:** 10.1038/s41598-021-93199-4

**Published:** 2021-07-07

**Authors:** Alexander Walton, Amy L. Toth, Adam G. Dolezal

**Affiliations:** 1 Department of Ecology, Evolution, and Organismal Biology, Iowa State University, Ames, IA USA; 2 Department of Entomology, Iowa State University, Ames, IA USA; 3 Department of Entomology, University of Illinois at Urbana-Champaign, Urbana, IL USA

**Keywords:** Viral infection, Gene expression, Ecophysiology

## Abstract

The consequences of early-life experiences are far reaching. In particular, the social and nutritional environments that developing animals experience can shape their adult phenotypes. In honeybees, larval nutrition determines the eventual social roles of adults as reproductive queens or sterile workers. However, little is known about the effects of developmental nutrition on important adult worker phenotypes such as disease resilience. In this study, we manipulated worker developmental nutrition in two distinct ways under semi-natural field conditions. In the first experiment, we restricted access to nutrition via social isolation by temporarily preventing alloparental care. In the second experiment, we altered the diet quality experienced by the entire colony, leading to adult bees that had developed entirely in a nutritionally restricted environment. When bees from these two experiments reached the adult stage, we challenged them with a common bee virus, Israeli acute paralysis virus (IAPV) and compared mortality, body condition, and the expression of immune genes across diet and viral inoculation treatments. Our findings show that both forms of early life nutritional stress, whether induced by lack of alloparental care or diet quality restriction, significantly reduced bees’ resilience to virus infection and affected the expression of several key genes related to immune function. These results extend our understanding of how early life nutritional environment can affect phenotypes relevant to health and highlight the importance of considering how nutritional stress can be profound even when filtered through a social group. These results also provide important insights into how nutritional stress can affect honeybee health on a longer time scale and its potential to interact with other forms of stress (i.e. disease).

## Introduction

Early-life experiences can have long lasting effects, and environmental variation during juvenile development can lead to morphological, physiological, and epigenomic changes that permanently alter adult phenotypes^1^. Nutrition in particular has received substantial focus as an aspect of the developmental or juvenile environment having far reaching consequences. Variation in nutrition during development can guide phenotypic plasticity, telegraphing cues to offspring that trigger adaptive responses to the environment^2^, and can even generate the production of vastly different adaptive adult phenotypes^3–5^. However, more extreme nutritional perturbations can lead to negative health consequences, with the early life nutritional environment implicated as a major contributor to disease risks^2,6^. Poor juvenile nutrition can also negatively affect survival^7–9^, reproduction^10^, immune function, and parasite resistance^11–17^, with impacts spanning generations^18–20^. In many of these examples, juvenile nutritional stress is filtered through other individuals, i.e., perturbations in diet are determined by parental care and resources; thus, the developmental environment is therefore an inherently social phenomenon. However, most studies focus on very small social groups, i.e., parent–offspring interactions, and we lack perspective on the social flow of nutrients in the context of larger social groups.

The honeybee, *Apis mellifera,* is a powerful system for studying how nutritional variation shapes adult phenotypes in the context of a large, complex social group. Honeybees have been used extensively to study how a nutritional switch, mediated by gene expression cascades and hormonal modulation, can produce dramatically different adult phenotypes, i.e., queens vs. workers^21–25^. Among workers, developmental environment can have important lifelong effects on foraging behavior^26^, aggression^27^, and cooperativeness^28^. The consequences of developmental nutrition on adult health are less understood, despite increasing attention on how nutrition affects immune response and pathogen resilience. Concerns about landscape simplification and reduced floral resources have led to hypotheses that reduced nutrient availability synergizes with increasingly widespread pathogen pressure, leading to increased morbidity and mortality^29–32^. An improved adult diet could mitigate these effects by maintaining immunocompetence^33^; for example, the detrimental effects of the microsporidian gut parasite *Nosema ceranae* can be offset by pollen quantity^34^, quality, and diversity^35^.

The most detrimental stressor managed honeybees currently face, however, is pressure from the parasitic mite *Varroa destructor*. These ectoparasites feed on the hemolymph and fat body of developing and adult bees^36^ and also vector a variety of highly detrimental viruses^37,38^. Nutrition has been shown to affect incidence of several of these viruses, including deformed wing virus (DWV)^39^ and black queen cell virus (BQCV). Adult diet can affect survivorship^29,40^ and transcriptional responses^41^ to infection with Israeli acute paralysis virus (IAPV). IAPV is a widespread virus^42^ that has been associated with large-scale colony losses^43^, and it produces distinct pathological phenotypes including shivering, paralysis, and death in a relatively short and repeatable window^44,45^. Although there is evidence that resilience to some viruses is heritable^86^, it is not clear how developmental nutrition affects adult phenotypes in response to virus challenge or how group-level nutritional differences (i.e., at the colony level) perturb individuals developing in these conditions. Here, we used experimental manipulations to test the hypothesis that developmental nutrition affects bees’ resilience to virus infection, and begin to explore the molecular underpinnings of these differences. To accomplish this, we used two different approaches to manipulate honeybee larval nutrition under semi-natural field conditions. First, we experimentally restricted larvae from receiving alloparental care for a short window during development, preventing bees from being fed and receiving other alloparental stimuli. Second, we manipulated colony-level diet by feeding experimental colonies only a high- or low-quality pollen source, producing colonies that experienced longer-term differential nutrition. In both cases, we predicted that the manipulations would be subtle enough to still allow production of seemingly-normal adult workers, but with increased sensitivity to infection, likely through modulation of immune responsiveness. We predicted that this would manifest in different levels of survivorship when faced with an IAPV challenge and alterations in the expression of immunity-related genes. We present evidence that both forms of stress significantly reduce bees’ resilience to virus infection. These findings have important ramifications to our understanding of how developmental nutrition affects pathogen responses, particularly within the complex network of environmental stressors faced by pollinators.

## Methods

### Experiment 1: short term larval starvation through restriction of alloparental care

**Figure 1:**
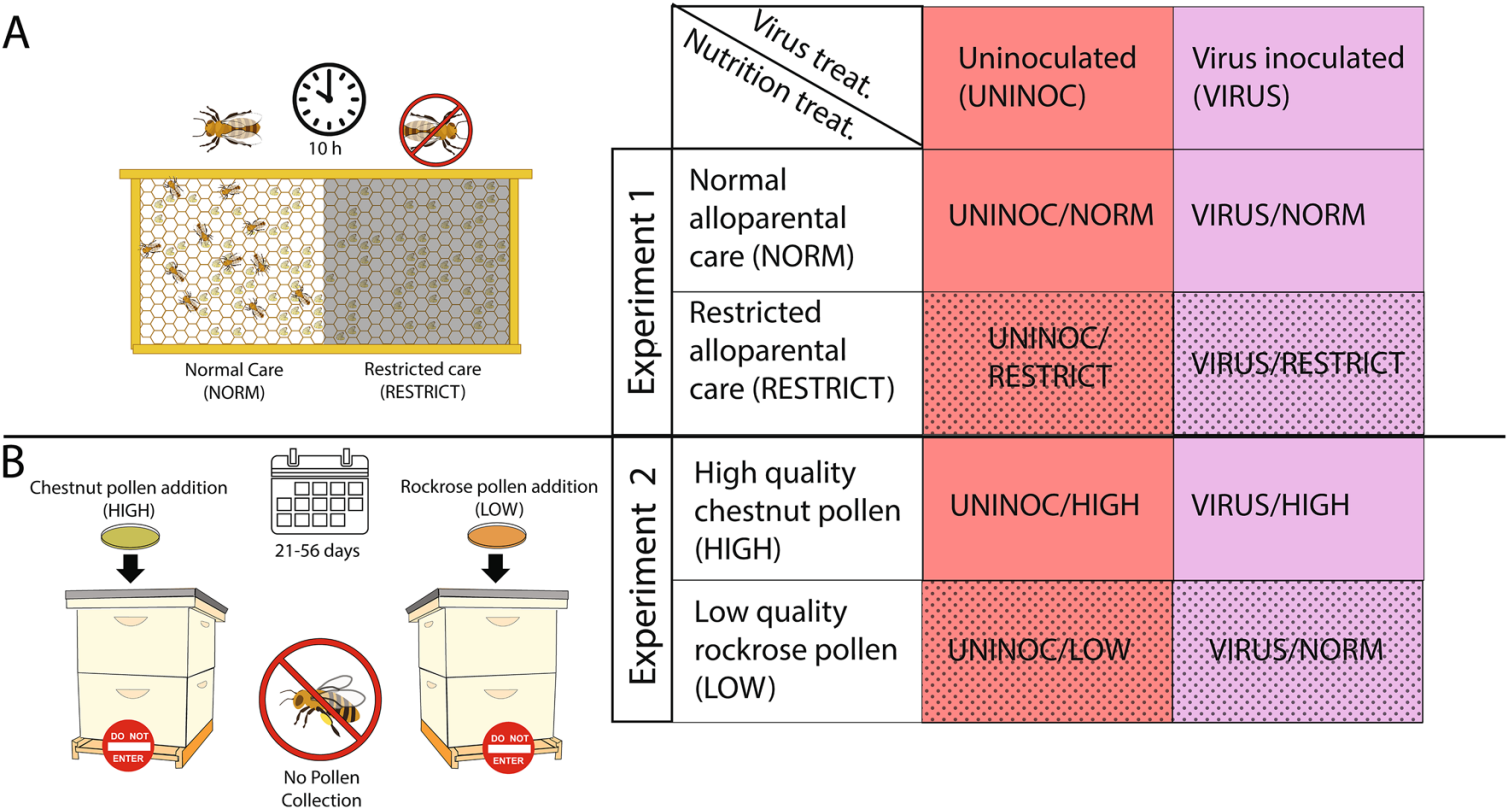
Graphical depiction of experimental treatments and table grid summarizing each treatment type. (**A**) In Experiment 1, adult workers were restricted from half of a frame (gray box over frame), preventing larvae from receiving alloparental care for a 10 h window (clock depicted), producing adult workers that experienced normal (NORM) or restricted (RESTRICT) care. (**B**) In Experiment 2, experimental bee hives were produced with equal adult bee populations; these colonies were fitted with pollen traps that prevented returning foragers from bringing environmental pollen into the hives. Instead, each hive was continuously fed a dish of pollen patty made from high-quality *Castanea sp.* (HIGH) or low-quality *Cistus sp.* (LOW) pollen. This treatment persisted over several generations of workers reared within the colonies (calendar depicted). As day-old adults, bees from each of the nutritional treatments were fed either a sterile solution (UNINOC) or an IAPV inoculum (VIRUS). In all figures, lower nutritional condition treatments are demarked with stippled fills; virus treatment is purple and uninoculated is pink.

First, we sought to produce honeybee adults that experienced a highly-standardized form of nutritional deprivation as larvae while still reared under mostly normal colony conditions. To do so, we used a protocol identical to that described in^28^, as modified from^46–48^. By physically restricting adult workers from interacting with developing larvae for a 10 h window during the 5th larval instar, we produced bees of two treatment types: those receiving normal alloparental care (denoted as NORM) and those experiencing restricted care (denoted as RESTRICT, Fig. 1). For detailed explanation of how these treatments were performed, see above references or the Additional Methods section of the Supplementary Material.

### Experiment 2: long term colony-level diet quality manipulation

While Experiment 1 allows a repeatable, standardized nutritional treatment, it is confounded by also preventing other nursing behaviors, such as grooming and cleaning, i.e., it starves the larvae nutritionally and socially. Therefore, we produced colony-level nutritional treatments by feeding experimental colonies pollen diets from single-source pollens that are both naturally collected by bees, but with different nutritional attributes—either high-quality *Castanea *sp. pollen (HIGH treatment) or low-quality *Cistus *sp. pollen (LOW treatment). These pollen sources differ in multiple key macro- and micronutrients and cause different responses to immune challenges to adults^29,35,41^. Experimental bees were those which had been reared by a preceeding generation that had experienced the nutritional treatment for their entire adult lives. For a highly detailed description of how these hives were constructed and maintained, see Additional Methods in the Supplementary Materials.

### Body quality analysis of nutritional treatments

For each nutritional treatment, we measured body mass and total lipid content of a subset of newly-emerged bees (pre-virus treatment) that were not used in the cage assays. Lipid content was assayed as described in^49^ as modified by^50^. See Additional Methods in the Supplementary Materials for a detailed description of lipid content assay.

### Cage assays

For both experiments, cages of newly-emerged bees from each treatment were assayed for mortality against a challenge with a virus inoculum previously shown to cause repeatable mortality due to infection primarily with Israeli acute paralysis virus (IAPV) using methods and inoculum identical to those described in^29^, as modified from^51^ and further described in^45^. Full details are provided in the Supplementary Material. In short, 35 newly-emerged bees derived from the treatment-specific mixture of bees were counted into an acrylic observation cage and treated with either sterile sucrose solution (hereafter the “UNINOC” treatment) or with a 1:1000 dilution of an IAPV virus inoculum (hereafter the “VIRUS” treatment). As described in^29^, one microliter of the 1:1000 dilution inoculum was estimated to contain 7.84 × 10^4^ IAPV. Experimental setup and treatment matrix shown in Fig. 1. The study was conducted according to the ARRIVE guidelines of animal research reporting^52^. The methods were carried out in accordance with the guidelines and regulations required for Biosafety level BSL-1, as outlined by the Iowa State University Office of Responsible Research, and were approved by the ISU Biosafety Committee.

### Virus titration and gene expression

We randomly-selected 10 cages from each treatment, pooled 6 bees collected from each cage at 36 h post-treatment, and extracted total body RNA using methods identical to^29^ and^53^. In short, RNA was extracted from each pooled sample using Trizol reagent followed by a DNAse treatment. Genome equivalents of IAPV were estimated for each sample via qPCR by calculation against the RNA standard curve developed by Carrillo-Tripp et al. 2016. Using the same RNA, we then measured gene expression via qPCR using the 2^−ΔΔCT^ method^54^ to calculate relative gene expression, with expression normalized to the internal control gene *actin*. *actin* expression was stable across our treatments (Experiment 1: ANOVA, p = 0.29; Experiment 2: ANOVA, p = 0.39). Expression data is shown relative to the “UNINOC/NORM” treatment as the reference group in Experiment 1 and the “UNINOC/HIGH” treatment in Experiment 2. We measured gene expression for five genes of interest: *cactus*, *hopscotch*, *hymenoptaecin*, *dicer*, and *vitellogenin*, each of which is known to play different important roles in insect immune response^55–59^. See Supplemental Table 1 for primer information.

### Statistical analysis

All analyses were performed in R using version 3.3.1^60^. For both experiments, mortality, virus titers, and gene expression were compared between the treatments via a linear mixed effect model using the lme function from the package nlme^61^; virus titers were first log transformed to meet normality assumptions and for biological relevance. Virus treatment, nutrition treatment, and virus*nutrition interaction were contained in each model as fixed effects, with ‘treatment generation’ as a random factor; in Experiment 2, hive source was also a random factor. We performed ANOVAs on these models, followed by a pairwise estimated marginal means (EMM) contrast with the emmeans function in the package emmeans using the Tukey HSD p-value correction^62^. Mass and lipid contents were compared using Welch’s t-test.

## Results

### Experiment 1

#### Body quality analysis

Bees from the RESTRICT group weighed significantly less than those from the NORM treatment (Welch’s t-test, t = 3.53, d.f. = 33.13, p = 0.0012; n_restricted_ = 16; n_normal_ = 20; Supplementary Fig. S1), but the proportion of their mass made up of lipids did not differ (Welch’s t-test, t = -2.16, d.f. = 7.27, p = 0.067; n_restricted_ = 6; n_normal_ = 6; Supplementary Fig. S1).

#### Virus challenge bioassay

**Figure 2:**
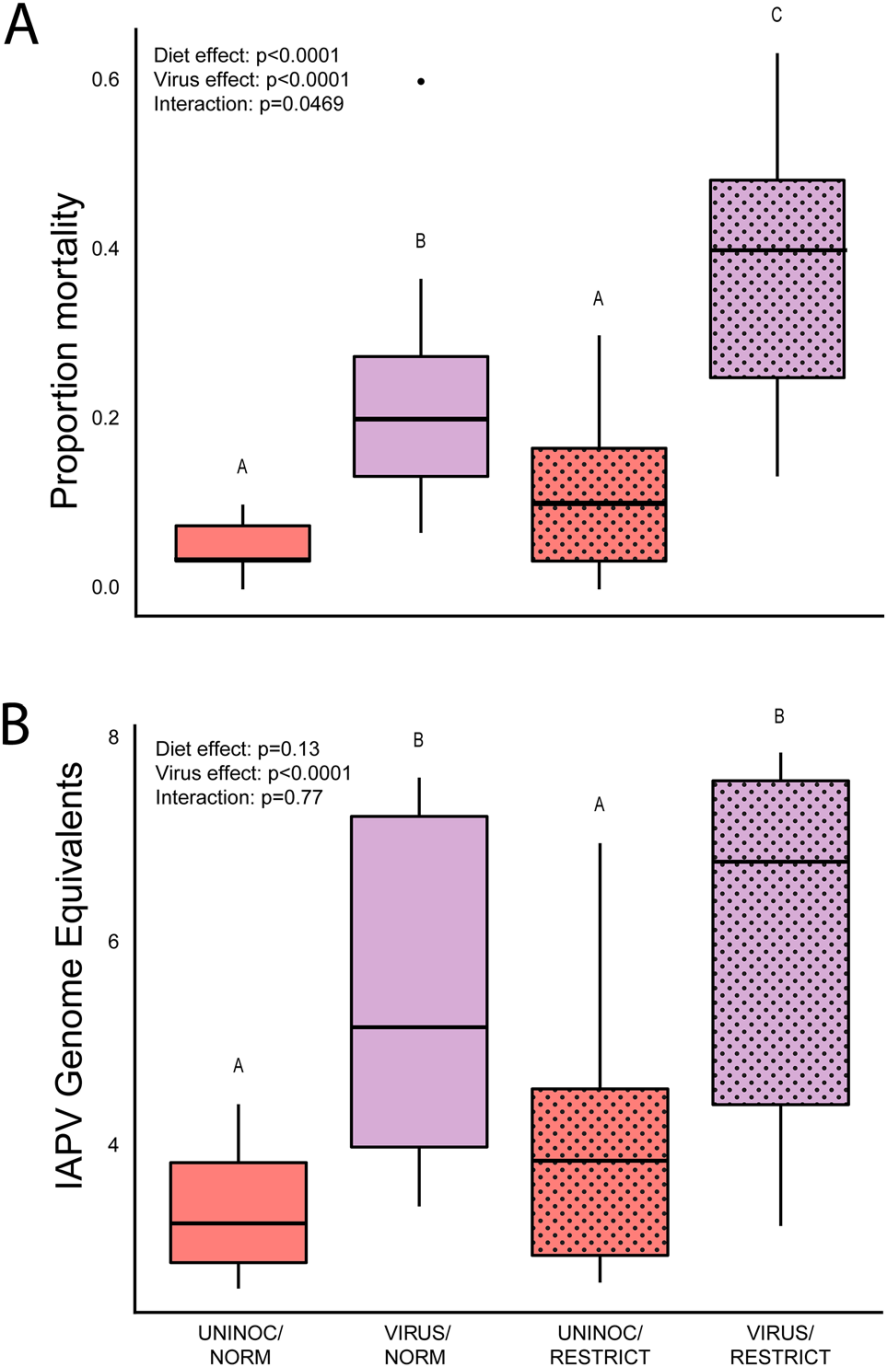
(**A**) Proportion mortality in cages of bees that experienced normal (NORM) or restricted (RESTRICT) conditions during development fed sterile sucrose (UNINOC) or virus inoculum (VIRUS) as adults, 96 h post inoculation (hpi); (**B**) Estimated genome equivalents, calculated against a standard curve, of IAPV in pooled samples from randomly-selected cages from each treatment. Boxplots display median, interquartile range, and full data range. Resulting p-values from 2-factor ANOVAs for each treatment and their interaction noted in top left; letters denote significant differences in posthoc comparisons of each group (ANOVA followed by Tukey HSD, p < 0.05; full statistical report in Supplemental Table 2).

Comparisons of the two virus (UNINOC vs. VIRUS) and two nutrition (NORM vs RESTRICT) factors revealed significant differences due to treatment in mortality (Fig. 2A; linear mixed effects model followed by two-factor ANOVA with interaction term; see Supplemental Table 2 for complete statistical results, sample sizes, and post-hoc contrasts). Virus-inoculated bees exhibited significantly higher mortality than uninoculated (ANOVA, p < 0.0001), restricted bees exhibited significantly higher mortality than normal (ANOVA, p < 0.0001), and there was a significant interaction between the two factors of nutrition and virus inoculation (ANOVA, p < 0.05). Pairwise contrasts revealed significant differences between the four combined treatment groups, with all groups different from each other (EMM, Tukey posthoc correction, p < 0.0001) except the two uninoculated treatments (EMM, Tukey posthoc correction, p = 0.31). Thus, while the two nutritional treatments did not differ from each other when the virus stimulus was absent, VIRUS/RESTRICT bees exhibited significantly higher mortality than both of the uninoculated groups and the VIRUS/NORM group, which exhibited significantly higher mortality than the uninoculated groups.

#### IAPV titers

We found significant differences due to experimental treatment on IAPV levels of bees collected 36 h into the assay (Fig. 2B; linear mixed effects model followed by two-factor ANOVA with interaction term; n = 10 per treatment; see Supplemental Table 2 for complete statistical results, and post-hoc contrasts). Virus-inoculated bees exhibited significantly higher titers than uninoculated bees (ANOVA, p < 0.0001). However, there were no significant differences by nutritional treatment (ANOVA, p = 0.13), and there was no significant interaction between the two factors (ANOVA, p = 0.77). Pairwise contrasts revealed that IAPV levels were not different between the uninoculated NORM and RESTRICT bees (EMM, Tukey posthoc correction, p > 0.05), and both showed lower IAPV levels than either virus-inoculated group (EMM, Tukey posthoc correction, p < 0.001). The two virus-inoculated treatment groups did not significantly differ from each other (EMM, Tukey posthoc correction, p > 0.05). Thus, overall, both virus-inoculated groups exhibited significantly elevated IAPV titers compared to the uninoculated, irrespective of the nutritional treatment.

#### Gene expression

**Figure 3:**
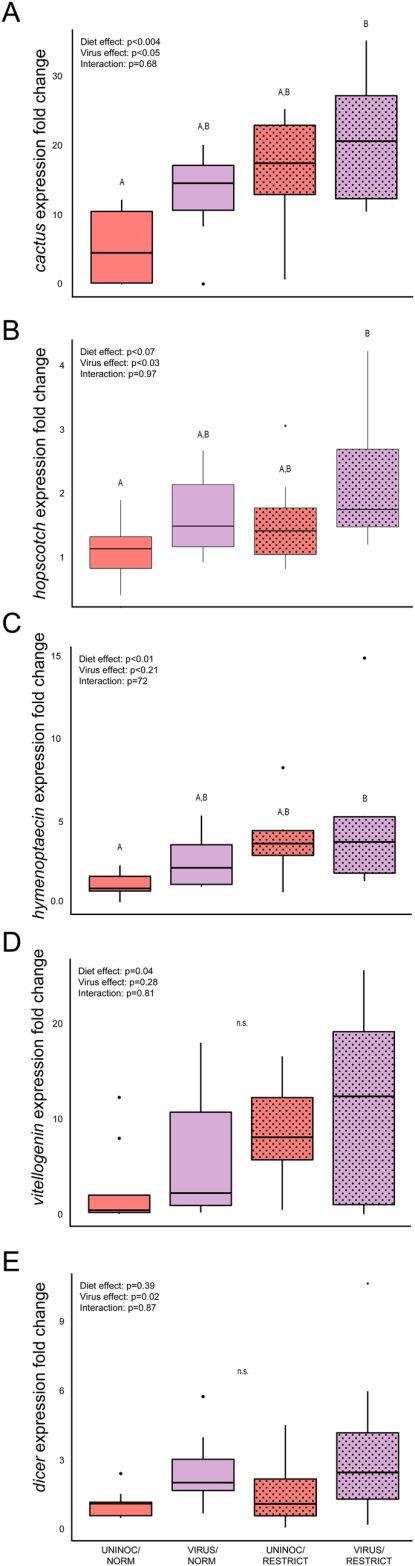
Immune gene expression of bees that either experienced normal (NORM) or restricted (RESTRICT) treatments during development and fed sterile sucrose (UNINOC) or virus inoculum (VIRUS) as adults. Boxplots display median, interquartile range, and full data range. Resulting p-values from 2-factor ANOVAs for each treatment and their interaction noted in top left of each plot; letters denote significant differences in posthoc comparisons of each group (ANOVA followed by Tukey HSD, p < 0.05; full statistical report in Supplemental Table 2). (**A**) *Cactus* expression was higher in bees from the VIRUS/RESTRICT treatment than those from the UNINOC/NORM treatment and had higher expression in RESTRICT vs NORM bees when averaged over viral treatments. (**B**) *Hopscotch* expression was higher in the VIRUS/RESTRIC than in the UNINOC/NORM treatment bees, and had higher expression in VIRUS groups when comparted to UNINOC groups when averaged over diet treatments. (**C**) *Vitellogenin* expression differed only when larval diet contrast results were averaged over viral treatment, showing higher expression in bees from the RESTRICT compared to the NORM treatments. (**D**) The expression of *dicer* was significantly different only when larval diet contrast results were averaged over viral treatment, showing higher expression RETRICT compared to NORM treatment bees.

Diet and viral treatments had varying effects on expression of the five immunity genes measured in this study (Fig. 3; see Supplemental Table 2 for complete statistical results, sample sizes, and post-hoc contrasts).

The gene *cactus* had significantly higher expression in RESTRICT vs NORM groups (Fig. 3A; ANOVA, p = 0.004). Post-hoc contrasts revealed *cactus* expression was higher in the VIRUS/RESTRICT treatment bees than UNINOC/NORM treatment bees (Tukey HSD, p = 0.005), but not significantly different between any other treatment comparisons.

The gene *hopscotch* also showed significantly higher expression in virus-inoculated groups compared to uninoculated (Fig. 3B; ANOVA, p = 0.03). Post-hoc contrasts revealed *hopscotch* expression was higher in the VIRUS/RESTRICT treatment bees compared to the UNINOC/NORM treatment bees (Tukey HSD_,_ p = 0.02).

*Hymenoptaecin* expression was higher in nutritionally restricted bees compared to bees reared under normal nutritional conditions (ANOVA, p = 0.01), but there were no significant differences by viral inoculation treatment (ANOVA, p = 0.21). Post-hoc contrasts showed that *hymenoptaecin* was more highly expressed in VIRUS/RESTRICT treatment bees than UNINOC/NORM treatment bees (Fig. 3C; Tukey HSD, p = 0.04).

*Vitellogenin* expression differed only when larval diet contrast results were averaged over viral treatment levels, showing higher expression in bees that were nutritionally restricted relative to normal (Fig. 3D, ANOVA, p = 0.04).

The expression of *dicer* was significantly different only when larval diet contrast results were averaged over viral treatment, showing higher expression in bees that were nutritionally restricted compared to normally reared bees (Fig. 3E, ANOVA, p = 0.02).

### Experiment 2

#### Body quality analysis

There were no significant differences in the mass or lipid content of bees from the HIGH and LOW pollen diet treatments (Welch’s t-test, p > 0.05; n_low_ = 12, n_high_ = 10, Supplemental Fig. S2).

#### Virus challenge bioassay

**Figure 4:**
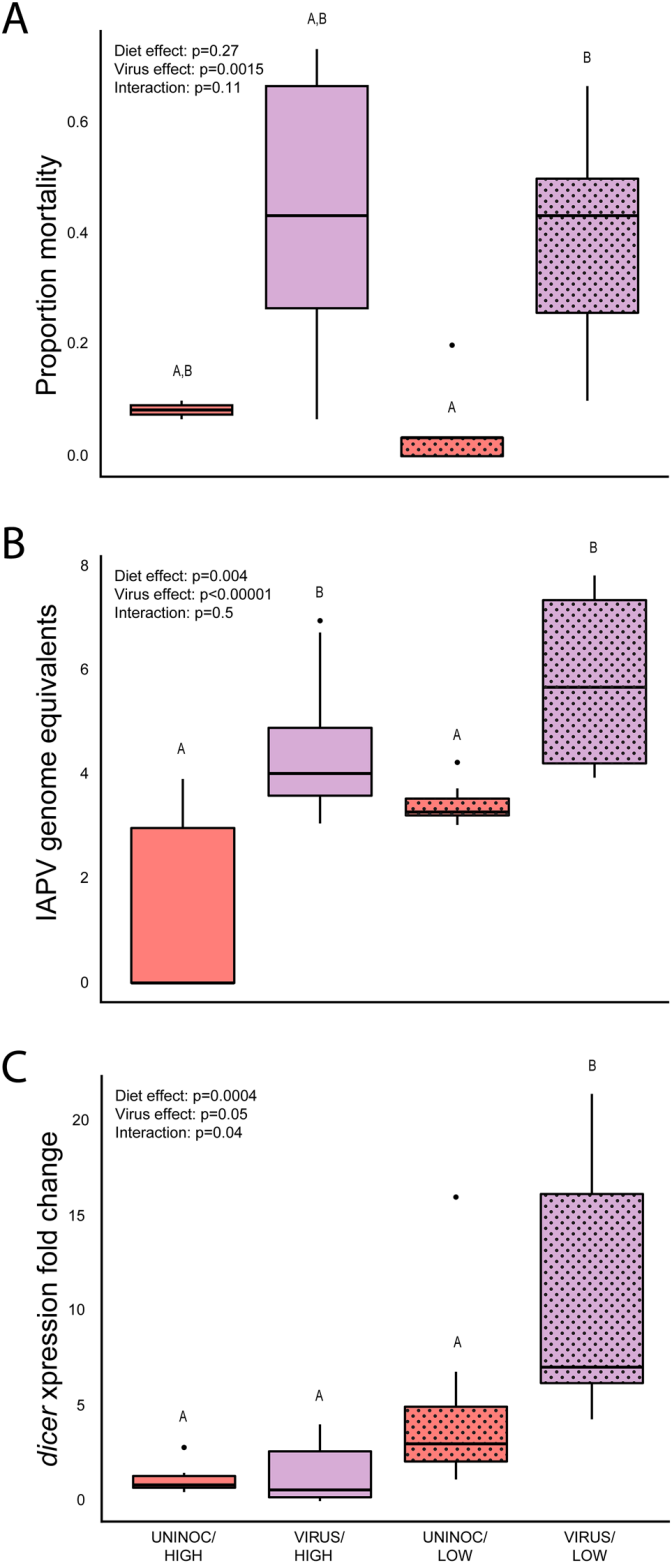
(**A**) Proportion mortality in cages of bees reared under LOW (*Cistus* diet) or HIGH (*Castanea* diet) conditions and fed sterile sucrose (UNINOC) or virus inoculum (VIRUS) as adults, 96 h post inoculation (hpi); (**B**) Estimated genome equivalents, calculated against a standard curve, of IAPV in pooled samples from randomly-selected cages from each treatment. (**C**) Expression of *dicer* in bees from each treatment, showing that both diet and virus treatment had significant effects on expression, with bees from the VIRUS/LOW group exhibiting significant higher expression than all other groups. Boxplots display median, interquartile range, and full data range. Resulting p-values from 2-factor ANOVAs for each treatment and their interaction noted in top left; letters denote significant differences in posthoc comparisons of each group (ANOVA followed by Tukey HSD, p < 0.05; full statistical report in Supplemental Table 3).

Comparisons of the two virus (IAPV inoculated vs. uninoculated) and two nutrition (high-quality vs. low-quality) factors revealed significant differences due to treatment in the proportion of bees that died during the 96 h assay (Fig. 4A; linear mixed effects model followed by two-factor ANOVA with interaction term; see Supplemental Table 3 for complete statistical results, sample sizes, and post-hoc contrasts), with significantly elevated mortality in the virus-inoculated groups (ANOVA, p < 0.0015), but no significant effect from the nutrition treatment alone (ANOVA, p = 0.27), and no significant interaction between the two factors (ANOVA, p < 0.11). Pairwise contrasts revealed significantly higher mortality in the VIRUS/LOW vs. UNINOC/LOW treatments (EMM, Tukey posthoc correction, p = 0.004), but no other significant differences (EMM, Tukey posthoc correction, p > 0.05). Overall, VIRUS/LOW bees thus exhibited significantly elevated mortality compared to UNINOC/HIGH bees, but VIRUS/HIGH bees were not significantly different from any treatments, i.e., they were intermediate.

#### IAPV titers

We found significant differences due to experimental treatment in IAPV levels of bees collected 36 h into the assay (Fig. 4B, linear mixed effects model followed by two-factor ANOVA with interaction term; See Supplemental Table 3 for complete statistical results, sample sizes, and post-hoc contrasts), with virus-inoculated bees exhibiting significantly higher titers than uninoculated bees (ANOVA, p < 0.0001), and bees from low-quality pollen-fed colonies showing significantly higher IAPV titers compared to bees from high-quality pollen-fed colonies (ANOVA, p = 0.004), but no significant interaction between the two factors (ANOVA, p = 0.50). Pairwise contrasts revealed that IAPV levels were significantly lower in the UNINOC/HIGH bees compared to both the VIRUS/HIGH (EMM, Tukey posthoc correction, p = 0.003) and VIRUS/LOW bees (EMM, Tukey posthoc correction, p < 0.0001). Within the LOW treatment groups, virus-inoculated bees exhibited significantly higher IAPV titers than their uninoculated counterparts (EMM, Tukey posthoc correction, p = 0.02). Thus, overall, VIRUS bees from both nutritional treatments exhibited elevated IAPV titers compared to their uninoculated counterparts, and LOW bees had higher IAPV titers than HIGH bees.

#### Gene expression

Diet and viral treatments had varying effects on expression of the immunity genes measured in this study (Fig. 4C, See Supplemental Table 3 for complete statistical results, sample sizes, and post-hoc contrasts).

The gene *dicer* had significantly higher expression in LOW bees than in HIGH bees, when averaged over viral treatment (Fig. 4C, mixed model ANOVA, p = 0.0004), there was a significant effect of viral treatment on *dicer* expression (mixed model ANOVA, p = 0.05), and there was a significant interaction effect of diet and viral treatments (ANOVA, p = 0.04). Post-hoc contrasts showed that *dicer* expression was higher in the LOW/VIRUS bees than in the other three treatments (see Supplemental Table 3 for statistical reports of all contrasts).

There were no significant effects of virus-inoculation or nutrition treatments on the expression of *cactus*, *hopscotch*, *hymenoptaecin*, or *vitellogenin* (see Supplemental Table 3 for statistical reports of all contrasts).

## Discussion

The nutritional environment during development can have long-term effects, modulating an individual’s adult phenotype and even impacting the fitness of offspring^18–20^. While these effects are often negative, affecting survivorship and disease risks, developmental stress can also play an important adaptive role, priming individuals for the challenges of their adult environment^2,6^. Few systems, however, offer the opportunity to study how nutritional differences are manifest through social feeding, considering how the filter of a large social group connects individual and group-level resource acquisition. In this study, we demonstrate how two different forms of socially-mediated developmental nutritional stress affect response to adult virus exposure and gene expression to affect a critical managed pollinator.

Restriction of alloparental care during development produced smaller workers with significantly greater susceptibility to infection-driven mortality. These differences likely represent realistic experiences, as lapses in a colony’s pollen resources can reduce alloparental care and larval nutrition^26,63^, and the differences we observed in adult mass are similar to those found due to natural comb cell size variation^64^. While there was no effect of nutritional treatment on baseline mortality, when inoculated with IAPV, a significant interaction between diet and virus treatment was observed, with workers from the RESTRICT treatment displaying approximately 15% higher mortality. While IAPV titers were higher in both inoculated groups compared to their uninoculated counterparts, the two inoculated groups did not exhibit differences from each other, suggesting that adult bees differed in their ability to tolerate virus infection, rather than reducing virus replication. However, because the restriction of alloparental care reduces larval nutrition along with other social interactions like grooming, these data do not allow us to disentangle the effects of nutritional stress from social isolation. Because social isolation has been shown to have negative consequences in a number of social species, from mammals to insects^65–67^, an important direction for future work will be to determine the relative contributions of nutrition and social stimulation on adult phenotypes.

Experiment 2 allowed us to begin addressing this challenge. Our colony-level pollen treatments affected developmental nutrition without directly manipulating alloparental care. While hives were experimentally produced, these experiments were performed in the field and likely represent realistic real-world conditions. Landscape-scale limitation of pollen resources has been hypothesized as a major driver in honeybee health declines^31^ and is associated with indicators of poor colony health, like reduced nurse bee lipid content^68–70^. Pollen diet has also been associated with health-related factors in adults, including lifespan^71,72^, immunocompetence^33^, and pathogen susceptibility^35,39,73^. Consistent with this previous work and Experiment 1, we found that rearing nutritional environment did not affect baseline survival, but IAPV inoculation resulted in significantly elevated mortality in bees from the LOW treatment colonies (*Cistus* sp.). There were, however, some differences between Experiments 1 and 2. VIRUS/LOW treatment bees experienced significantly higher mortality compared to either uninoculated diet treatment. VIRUS/HIGH treatment bees, however, exhibited intermediate mortality, not differing between the other groups. This is supported by the significant effect of diet treatment on IAPV titers, with bees from the HIGH group exhibiting lower levels, suggesting that the bees with higher quality larval nutrition may be reducing viral replication, keeping IAPV titers lower. These results are consistent with previous work showing that *Castanea sp.* pollen (HIGH) improves adult bee resilience against *N. ceranae*^35^ and IAPV^29^. Further, while restricted alloparental care in Experiment 1 produced less massive adults, colony-level nutritional treatment did not affect adult lipid proportion. Because the colonies in Experiment 2 had ad libitum access to their pollen treatments, nurse bees had the potential to adjust the quantity of food they feed to brood to compensate for low-quality nutrition. Further, the dietary factors that are required to produce bees of a given mass may not be the same as those involved in improving immune responses. For example, while the low-quality (*Cistus *sp.) and high-quality (*Castanea *sp.) pollens differ in many components, including protein content^35^, which likely affects mass, the *Cistus* pollen also has a lower concentration of trace micronutrients, including calcium and iron^29^. These micronutrients may be crucial for pathogen resistance^74^, and there has been growing interest in better understanding how they affect bee biology^75^.

In both experiments, virus treatment and diet manipulation were marked by changes in expression of genes associated with immune response, as well genes associated with other functions. As reported in prior studies examining gene expression in response to virus-diet alterations in honeybees^41^, different genes showed different responses to each form of stress and their interaction. In Experiment 1, regardless of nutritional treatment, challenge with IAPV resulted in higher expression of *dicer* and *hopscotch*. In Experiment 2, LOW pollen treatment resulted in higher expression of *dicer*, and bees inoculated with IAPV had higher expression of *dicer* than uninoculated bees. In fact, bees from the VIRUS/LOW treatment exhibited higher expression of *dicer* than all other treatments in Experiment 2. Dicer is an enzyme that is part of the RNA-interference pathway, a highly conserved system that identifies and combats RNA viruses^58^, and Hopscotch is a component of the JAK/STAT signaling pathway that is associated with honeybee immunity^56^. Further, in Experiment 1, *cactus* and *hymenoptaecin* were upregulated in RESTRICT bees, regardless of viral treatment. The Cactus protein is a component of the Toll immunity signaling pathway, which exhibits antimicrobial activity in honeybees^55^, and hymenoptaecin is an antimicrobial peptide involved in honeybee immune response to bacteria and viruses^57^. Overall, while the expression of these genes differs between the experiments, the patterns confirm that our inoculation treatments affected the expression of canonical immune genes and shows that different forms of developmental perturbations result in different gene expression outcomes later in life.

In both experiments, virus infection caused expression differences of some immune genes regardless of diet treatment, and the genes affected differed between diet experiments, reaffirming that our treatments produced fundamentally different forms of developmental stress. As expected, virus infection caused an upregulation of genes associated with the immune system. However, the type of juvenile nutritional environment affected how the immune system responded to infection (via gene expression change), and subsequently, the immune system’s efficacy at successfully staving off the infection. In our study, virus infection caused higher immune gene expression differences between workers from the RESTRICT and NORM diet treatments than between workers from LOW and HIGH colony-level pollen diet treatments. This suggests that the long-term effects of acute larval starvation may be more pronounced than differences in larval diet quality.

In addition, in Experiment 1, *vitellogenin* expression was upregulated in RESTRICT bees, regardless of virus treatment. On the surface, this result may seem counterintuitive, as vitellogenin acts as a storage protein and plays a role in immunity^59^. However, upregulation of *vitellogenin* has also been implicated as a hormetic response to stress. Hormesis occurs when stress induces mechanisms to protect an individual against continuing or future stress^76^. For example, dietary restriction can result in increased lifespan in many animals, including honeybees^77^. In *Bombyx mori* moths, heat stress during pupal development causes upregulation of *vitellogenin*^78^, presumably because of its broad protective properties^59^. Additionally, when honeybee colonies are exposed to the insecticide imidacloprid in the field, worker bees show increased expression of *vitellogenin*^87^, further supporting the potential for *vitellogenin* as an important part of hormesis. This is consistent with the upregulation in *vitellogenin* that we observed in bees from the RESTRICT treatments. While this did not affect IAPV replication or mortality, it may prove adaptive in response to other factors. In any case, this result also suggests that, as in many other animals, the responses to developmental nutritional stresses can result in adaptive benefits under some conditions^2^.

Together, our findings provide valuable insights into how the juvenile environment affects adult phenotypes and have ramifications for our understanding of how nutrition affects the world’s most important managed pollinator. By manipulating the social nutritional experiences of larvae, we expand our understanding of how early-life environments can result in long-term effects on critical phenotypes^79–81^ like disease resistance. Further, these results have important consequences for our understanding of honeybee nutrition. Many recent studies have shown the importance of nutritional availability, both in laboratory^29,35,75^ and field studies^70,73,82–85^. While these have provided insights into the importance of the nutritional landscape for colonies or the effects of different diets under controlled conditions, most have had little, if any, connection to the diet of larvae. With the system described here, future work can investigate how individuals reared under different social and nutritional developmental conditions respond to other stimuli and how the environmental conditions filter through a complex social group.

## Supplementary Information

Supplementary Information.

## Data Availability

Data available from the Dryad Digital Repository.
