# Supplementary material for: Developmental environment shapes honeybee worker response to virus infection
Source: Sci Rep. 2021 Jul 7;11:13961. doi: 10.1038/s41598-021-93199-4 (PMC8263599; doi:10.1038/s41598-021-93199-4)
Supplement: Supplementary file 1 — Supplementary Information. [file 41598_2021_93199_MOESM1_ESM.docx]

Supplemental material for:

**Developmental environment shapes honeybee worker response to virus infection**

Alexander Walton (1), Amy L. Toth (1,2), Adam G. Dolezal (3)

1. Department of Ecology, Evolution, and Organismal Biology, Iowa State University, Ames, Iowa, USA
2. Department of Entomology, Iowa State University, Ames, Iowa, USA
3. Department of Entomology, University of Illinois, Champaign-Urbana, Illinois, USA

**Supplemental Figure S1**: A) Mass and B) proportion lipid content of bees from Experiment 1, reared under normal (NORM) and restricted (RESTRICT) conditions. Boxplots display median, interquartile range, and full data range. Different letters above bars denote significant differences (Welch’s t-test, p<0.05).

| **Gene** | **Forward primer** | **Reverse primer** | **Source** | **Gene ID**  **(accession number or Honey Bee Genome Project gene name)** |
| --- | --- | --- | --- | --- |
| *vitellogenin* | GTTGGAGAGCAACATGCAGA | TCGATCCATTCCTTGATGGT | Wang, et al. (2012) | AJ517411.1 |
| *hymenoptaecin* | CTCTTCTGTGCCGTTGCATA | GCGTCTCCTGTCATTCCATT | Evans, et al. (2006) | GB17538 |
| *apidaecin* | TAGTCGCGGTATTTGGGAAT | TTTCACGTGCTTCATATTCTTCA | Evans, et al. (2006) | GB17782 |
| *cactus* | CACAAGATCTGGAGCAACGA | GCATTCTTGAAGGAGGAACG | Evans, et al. (2006) | GB10655 |
| *hopscotch* | ATTCATGGCATCGTGAACAA | CTGTGGTGGAGTTGTTGGTG | Evans, et al. (2006) | GB12159 |
| *dicer* | CCAACAGGAGCTGGAAAAAC | TCTCCACTAAGTGCTGCACAA | Galbraith et al. (2015) | XM_006571316.1 |
| *actin* | TGCCAACACTGTCCTTTCTG | AGAATTGACCCACCAATCCA | Cristino, et al. (2014) | NM_001185145.1 |

**Supplemental Table 1:** Immune gene primers.

Primer full references:

Cristino, A.S., Barchuk, A.R., Freitas, F.C., Narayanan, R.K., Biergans, S.D., Zhao, Z., Simoes, Z.L., Reinhard, J. and Claudianos, C. Neuroligin-associated microRNA-932 targets actin and regulates memory in the honeybee. *Nature Communications* (2014) doi: 10.1038/ncomms6529.

Evans, J.D., Aronstein, K., Chen, Y.P., Hetru, C., Imler, J.L., Jiang, H., Kanost, M., Thompson, G.J., Zou, Z. and Hultmark, D. Immune pathways and defence mechanisms in honey bees *Apis mellifera*. *Insect Molecular Biology* (2006) doi:10.1111/j.1365-2583.2006.00682.x.

Galbraith, D. A., Yang, X., Nino, E. L., Yi, S., & Grozinger, C. Parallel epigenomic and transcriptomic responses to viral infection in honey bees (*Apis mellifera*). *PLoS Pathogens* (2015) doi:10.1371/journal.ppat.1004713.

Wang Y., Brent C.S., Fennern E., Amdam G.V. Gustatory perception and fat body energy metabolism are jointly affected by vitellogenin and juvenile hormone in honey bees. *PLoS Genetics* (2012) doi:10.1371/journal.pgen.1002779

| **Mortality: linear mixed-effects model**  Type III ANOVA table from Satterwaite’s method | | | | | | | | | | | | | | |
| --- | --- | --- | --- | --- | --- | --- | --- | --- | --- | --- | --- | --- | --- | --- |
| Fixed effect | | numDF | | | denDF | | | | F-value | | | | p-value | |
| **Diet treatment** | | 1 | | | 74 | | | | 19.71 | | | | **<0.0001** | |
| **Viral treatment** | | 1 | | | 74 | | | | 254.23 | | | | **<0.0001** | |
| **Interaction** | | 1 | | | 74 | | | | 4.08 | | | | **0.0469** | |
| **Mortality: pairwise comparisons for all treatments**  Degrees-of-freedom method: Kenward-Roger  p-value adjustment: Tukey HSD | | | | | | | | | | | | | | |
| \| Sample sizes \| Contrast \| \| Estimate \| df \| t-ratio \| p-value \| \| --- \| --- \| --- \| --- \| --- \| --- \| --- \| \| Treatment 1 \| Treatment 2 \| \| n_NORM/UNINOC_ =20 n_NORM/VIRUS_ =20  n_RESTRICT/UNINOC_ =20 n_RESTRICT/VIRUS_ =19 \| **NORM/UNINOC** \| **NORM/VIRUS** \| -0.18 \| 74 \| -5.43 \| **<0.0001** \| \| **NORM/UNINOC** \| **RESTRICT/VIRUS** \| -0.33 \| 74 \| -9.91 \| **<0.0001** \| \| NORM/UNINOC \| RESTRICT/UNINOC \| -0.06 \| 74 \| -1.74 \| 0.31 \| \| **NORM/VIRUS** \| RESTRICT/VIRUS \| -0.15 \| 74 \| -4.56 \| **0.0001** \| \| **RESTRICT/UNINOC** \| NORM/VIRUS \| -0.12 \| 74 \| -3.69 \| **0.002** \| \| **RESTRICT/UNINOC** \| RESTRICT/VIRUS \| -0.27 \| 74 \| -8.19 \| **<0.0001** \| | | | | | | | | | | | | | | |
| **IAPV titers: linear mixed-effects model**  Type III ANOVA table from Satterwaite’s method | | | | | | | | | | | | | | |
| \| Fixed effect \| numDF \| denDF \| F-value \| p-value \| \| --- \| --- \| --- \| --- \| --- \| \| **Diet treatment** \| 1 \| 35 \| 2.41 \| 0.13 \| \| **Viral treatment** \| 1 \| 35 \| 35.83 \| **<0.0001** \| \| Interaction \| 1 \| 35 \| 0.09 \| 0.77 \| | | | | | | | | | | | | | | |
| **IAPV titers: pairwise comparisons for all treatments**  Degrees-of-freedom method: Kenward-Roger  p-value adjustment: Tukey HSD | | | | | | | | | | | | | | |
| \| Sample sizes \| Contrast \| \| Estimate \| df \| t-ratio \| p-value \| \| --- \| --- \| --- \| --- \| --- \| --- \| --- \| \| Treatment 1 \| Treatment 2 \| \| n_NORM/UNINOC_ =10 n_NORM/VIRUS_ =10  n_RESTRICT/UNINOC_ =10 n_RESTRICT/VIRUS_ =10 \| **NORM/UNINOC** \| **NORM/VIRUS** \| -3.01 \| 35 \| -4.81 \| **0.0002** \| \| **NORM/UNINOC** \| **RESTRICT/VIRUS** \| -3.51 \| 35 \| -5.60 \| **<0.0001** \| \| NORM/UNINOC \| RESTRICT/UNINOC \| -0.74 \| 35 \| -1.31 \| 0.56 \| \| NORM/VIRUS \| RESTRICT/VIRUS \| -0.5 \| 35 \| -0.89 \| 0.81 \| \| **RESTRICT/UNINOC** \| **NORM/VIRUS** \| -2.27 \| 35 \| -3.63 \| **0.005** \| \| **RESTRICT/UNINOC** \| **RESTRICT/VIRUS** \| -2.77 \| 35 \| -4.42 \| **0.0005** \| | | | | | | | | | | | | | | |
| **Immune gene expression: linear mixed-effects model**  Type III ANOVA table from Satterwaite’s method | | | | | | | | | | | | | | |
| Gene | | Fixed effect | | numDF | | denDF | F-value | | | | p-value | | | |
| *Cactus* | | **Diet treatment** | | 1 | | 23.0 | 10.26 | | | | **0.004** | | | |
|  |  | Viral treatment | | 1 | | 23.2 | 4.27 | | | | 0.05 | | | |
|  |  | Interaction | | 1 | | 23.23 | 0.178 | | | | 0.68 | | | |
| *Dicer* | | Diet treatment | | 1 | | 31 | 0.73 | | | | 0.39 | | | |
|  |  | **Viral treatment** | | 1 | | 31 | 5.59 | | | | **0.02** | | | |
|  |  | Interaction | | 1 | | 21 | 0.03 | | | | 0.87 | | | |
| *Hopscotch* | | Diet treatment | | 1 | | 30 | 3.43 | | | | 0.07 | | | |
|  |  | **Viral treatment** | | 1 | | 30 | 5.53 | | | | **0.03** | | | |
|  |  | Interaction | | 1 | | 30 | 0.001 | | | | 0.97 | | | |
| *Hymenoptaecin* | | **Diet treatment** | | 1 | | 31 | 7.04 | | | | **0.01** | | | |
|  |  | Viral treatment | | 1 | | 31 | 1.62 | | | | 0.21 | | | |
|  |  | Interaction | | 1 | | 31 | 0.13 | | | | 0.72 | | | |
| *Vitellogenin* | | **Diet treatment** | | 1 | | 29 | 4.39 | | | | **0.04** | | | |
|  |  | Viral treatment | | 1 | | 29 | 1.23 | | | | 0.28 | | | |
|  |  | Interaction | | 1 | | 29 | 0.06 | | | | 0.81 | | | |
| **Immune gene expression: pairwise comparisons for all treatments**  Degrees-of-freedom method: Kenward-Roger  p-value adjustment: Tukey HSD | | | | | | | | | | | | | | |
| Gene | Sample sizes | | Contrast | | | | | Estimate | | df | | t-ratio | | p-value |
|  |  |  | Treatment 1 | | | Treatment 2 | |  |  |  |  |  |  |  |
| *Cactus* | n_NORM_*_/_*_UNINOC_ =9 n_NORM_*_/_*_VIRUS_ =6  n_VIRUS_*_/_*_UNINOC_ =7 n_VIRUS_*_/_*_VIRUS_ =6 | | NORM/UNINOC | | | NORM*/*VIRUS | | -7.3 | | 24 | | -1.84 | | 0.27 |
|  |  |  | NORM*/*UNINOC | | | RESTRICT*/*UNINOC | | -10.59 | | 23 | | -2.60 | | 0.07 |
|  |  |  | **NORM/UNINOC** | | | **RESTRICT*/*VIRUS** | | -15.40 | | 23 | | -3.79 | | **0.005** |
|  |  |  | NORM/VIRUS | | | RESTRICT*/*UNINOC | | -3.32 | | 23 | | -0.78 | | 0.86 |
|  |  |  | NORM/VIRUS | | | RESTRICT*/*VIRUS | | -8.13 | | 23 | | -1.91 | | 0.25 |
|  |  |  | RESTRICT/UNINOC | | | RESTRICT*/*VIRUS | | -4.81 | | 23 | | -1.09 | | 0.69 |
| *Dicer* | n_NORM_*_/_*_UNINOC_ =9 n_NORM_*_/_*_VIRUS_ =9  n_RESTRICT_*_/_*_UNINOC_ =8 n_RESTRICT_*_/_*_VIRUS_ =9 | | NORM/UNINOC | | | NORM*/*VIRUS | | -1.5 | | 30 | | -1.58 | | 0.40 |
|  |  |  | NORM*/*UNINOC | | | RESTRICT*/*UNINOC | | -0.47 | | 30 | | -0.48 | | 0.96 |
|  |  |  | NORM/UNINOC | | | RESTRICT*/*VIRUS | | -2.20 | | 30 | | -2.31 | | 0.12 |
|  |  |  | NORM/VIRUS | | | RESTRICT*/*UNINOC | | 1.03 | | 30 | | -2.31 | | 0.72 |
|  |  |  | NORM/VIRUS | | | RESTRICT*/*VIRUS | | -0.69 | | 30 | | -0.73 | | 0.88 |
|  |  |  | RESTRICT/UNINOC | | | RESTRICT*/*VIRUS | | -1.73 | | 30 | | -1.76 | | 0.31 |
| *Hopscotch* | n_NORM_*_/_*_UNINOC_ =9 n_NORM_*_/_*_VIRUS_ =9  n_RESTRICT_*_/_*_UNINOC_ =7 n_RESTRICT_*_/_*_VIRUS_ =9 | | NORM/UNINOC | | | NORM*/*VIRUS | | -0.59 | | 29 | | -1.69 | | 0.34 |
|  |  |  | NORM*/*UNINOC | | | RESTRICT*/*UNINOC | | -0.47 | | 29 | | -1.24 | | 0.61 |
|  |  |  | **NORM/UNINOC** | | | **RESTRICT*/*VIRUS** | | -1.08 | | 29 | | -3.08 | | 0.**02** |
|  |  |  | NORM/VIRUS | | | RESTRICT*/*UNINOC | | 0.13 | | 29 | | 0.34 | | 0.99 |
|  |  |  | NORM/VIRUS | | | RESTRICT*/*VIRUS | | -0.49 | | 29 | | -1.38 | | 0.52 |
|  |  |  | RESTRICT/UNINOC | | | RESTRICT*/*VIRUS | | -0.62 | | 29 | | -1.63 | | 0.38 |
| *Hymenoptaecin* | n_NORM_*_/_*_UNINOC_ =9 n_NORM_*_/_*_VIRUS_ =9  n_RESTRICT_*_/_*_UNINOC_ =8 n_RESTRICT_*_/_*_VIRUS_ =9 | | NORM/UNINOC | | | NORM*/*VIRUS | | -1.40 | | 30 | | -1.17 | | 0.65 |
|  |  |  | NORM*/*UNINOC | | | RESTRICT*/*UNINOC | | -2.59 | | 30 | | -2.09 | | 0.18 |
|  |  |  | **NORM/UNINOC** | | | **RESTRICT*/*VIRUS** | | -3.38 | | 30 | | -2.82 | | **0.04** |
|  |  |  | NORM/VIRUS | | | RESTRICT*/*UNINOC | | -1.19 | | 30 | | -0.96 | | 0.77 |
|  |  |  | NORM/VIRUS | | | RESTRICT*/*VIRUS | | -1.98 | | 30 | | -1.65 | | 0.37 |
|  |  |  | RESTRICT/UNINOC | | | RESTRICT*/*VIRUS | | -0.79 | | 30 | | -0.64 | | 0.92 |
| *Vitellogenin* | n_NORM_*_/_*_UNINOC_ =9 n_NORM_*_/_*_VIRUS_ =7  n_RESTRICT_*_/_*_UNINOC_ =8 n_RESTRICT_*_/_*_VIRUS_ =9 | | NORM/UNINOC | | | NORM*/*VIRUS | | -3.48 | | 29 | | -0.94 | | 0.78 |
|  |  |  | NORM*/*UNINOC | | | RESTRICT*/*UNINOC | | -6.01 | | 29 | | -1.67 | | 0.35 |
|  |  |  | NORM/UNINOC | | | RESTRICT*/*VIRUS | | -8.22 | | 29 | | -2.39 | | 0.10 |
|  |  |  | NORM/VIRUS | | | RESTRICT*/*UNINOC | | -2.52 | | 29 | | -0.67 | | 0.91 |
|  |  |  | NORM/VIRUS | | | RESTRICT*/*VIRUS | | -4.73 | | 29 | | -1.28 | | 0.58 |
|  |  |  | RESTRICT/UNINOC | | | RESTRICT*/*VIRUS | | -2.21 | | 29 | | -0.62 | | 0.92 |

**Supplemental Table 2:** Statistical analyses for Experiment 1. Significant results are in bold.

**Supplemental Figure S2**: A) Mass and B) proportion lipid content of bees from Experiment 2, reared in colonies fed only *Cistus* (HIGH) and *Castanea* (HIGH) nutritioanl conditions. Boxplots display median, interquartile range, and full data range. No groups exhibit significant differences.

|  | | | | | | | | | | | | | | |
| --- | --- | --- | --- | --- | --- | --- | --- | --- | --- | --- | --- | --- | --- | --- |
| **Mortality: linear mixed-effects model**  Type III ANOVA table from Satterwaite’s method | | | | | | | | | | | | | | |
| Fixed effect | | numDF | | | denDF | | | | F-value | | | | p-value | |
| Pollen diet | | 1 | | | 7 | | | | 1.46 | | | | 0.27 | |
| **Viral treatment** | | 1 | | | 52 | | | | 11.18 | | | | **0.0015** | |
| Interaction | | 1 | | | 52 | | | | 2.59 | | | | 0.11 | |
| **Mortality: pairwise comparisons for all treatments**  Degrees-of-freedom method: containment  p-value adjustment: Tukey HSD | | | | | | | | | | | | | | |
| \| Sample sizes \| Contrast \| \| Estimate \| df \| t-ratio \| p-value \| \| --- \| --- \| --- \| --- \| --- \| --- \| --- \| \| Treatment 1 \| Treatment 2 \| \| n_HIGH/UNINOC_ =11 n_HIGH/VIRUS_ =11  n_LOW/UNINOC_ =21 n_LOW/VIRUS_ =22 \| HIGH/UNINOC \| HIGH/VIRUS \| -0.03 \| 52 \| -0.64 \| 0.92 \| \| HIGH/UNINOC \| LOW/VIRUS \| -0.15 \| 7 \| -2.21 \| 0.21 \| \| HIGH/UNINOC \| LOW/UNINOC \| -0.03 \| 7 \| -0.44 \| 0.97 \| \| HIGH/VIRUS \| LOW/VIRUS \| -0.117 \| 7 \| -1.76 \| 0.36 \| \| HIGH/VIRUS \| LOW/UNINOC \| 0.0005 \| 7 \| 0.008 \| 1 \| \| **LOW/VIRUS** \| **LOW/UNINOC** \| -0.12 \| 52 \| -3.63 \| **0.004** \| | | | | | | | | | | | | | | |
| **IAPV titers: linear mixed-effects model**  Type III ANOVA table from Satterwaite’s method | | | | | | | | | | | | | | |
| \| Fixed effect \| numDF \| denDF \| F-value \| p-value \| \| --- \| --- \| --- \| --- \| --- \| \| **Pollen diet** \| 1 \| 28 \| 9.85 \| **0.004** \| \| **Viral treatment** \| 1 \| 28 \| 26.23 \| **1.989E-5** \| \| Interaction \| 1 \| 28 \| 0.46 \| 0.50 \| | | | | | | | | | | | | | | |
| **IAPV titers: pairwise comparisons for all treatments**  Degrees-of-freedom method: Kenward-Roger  p-value adjustment: Tukey HSD | | | | | | | | | | | | | | |
| \| Sample sizes \| Contrast \| \| Estimate \| df \| t-ratio \| p-value \| \| --- \| --- \| --- \| --- \| --- \| --- \| --- \| \| Treatment 1 \| Treatment 2 \| \| n_HIGH/UNINOC_ =7 n_HIGH/VIRUS_ =8  n_LOW/UNINOC_ =7 n_LOW/VIRUS_ =10 \| **HIGH/UNINOC** \| **HIGH/VIRUS** \| -3.08 \| 27.2 \| -3.88 \| **0.003** \| \| **HIGH/UNINOC** \| **LOW/VIRUS** \| -4.39 \| 27.4 \| -5.48 \| **<0.0001** \| \| HIGH/UNINOC \| LOW/UNINOC \| -2.03 \| 27.7 \| -2.34 \| 0.11 \| \| HIGH/VIRUS \| LOW/VIRUS \| -1.31 \| 26.5 \| -1.82 \| 0.29 \| \| HIGH/VIRUS \| LOW/UNINOC \| -1.05 \| 26.4 \| -1.34 \| 0.54 \| \| **LOW/VIRUS** \| **LOW/UNINOC** \| -2.36 \| 25.7 \| -3.19 \| **0.02** \| | | | | | | | | | | | | | | |
| **Immune gene expression: linear mixed-effects model**  Type III ANOVA table from Satterwaite’s method | | | | | | | | | | | | | | |
| Gene | | Fixed effect | | numDF | | denDF | F-value | | | | p-value | | | |
| *Cactus* | | Pollen diet | | 1 | | 27 | 3.35 | | | | 0.08 | | | |
|  |  | Viral treatment | | 1 | | 27 | 0.05 | | | | 0.82 | | | |
|  |  | Interaction | | 1 | | 27 | 0.25 | | | | 0.62 | | | |
| *Dicer* | | **Pollen diet** | | 1 | | 25.6 | 16.44 | | | | **0.0004** | | | |
|  |  | **Viral treatment** | | 1 | | 23.9 | 4.10 | | | | **0.05** | | | |
|  |  | **Interaction** | | 1 | | 23.8 | 4.78 | | | | **0.04** | | | |
| *Hopscotch* | | Pollen diet | | 1 | | 25.6 | 0.50 | | | | 0.48 | | | |
|  |  | Viral treatment | | 1 | | 24.4 | 1.79 | | | | 0.19 | | | |
|  |  | Interaction | | 1 | | 24.3 | 0.01 | | | | 0.91 | | | |
| *Hymenoptaecin* | | Pollen diet | | 1 | | 24.8 | 0.42 | | | | 0.52 | | | |
|  |  | Viral treatment | | 1 | | 22.7 | 0.24 | | | | 0.63 | | | |
|  |  | Interaction | | 1 | | 23.0 | 0.95 | | | | 0.34 | | | |
| *Vitellogenin* | | Pollen diet | | 1 | | 26 | 3.53 | | | | 0.07 | | | |
|  |  | Viral treatment | | 1 | | 26 | 0.02 | | | | 0.88 | | | |
|  |  | Interaction | | 1 | | 26 | 0.01 | | | | 0.93 | | | |
| **Immune gene expression: pairwise comparisons for all treatments**  Degrees-of-freedom method: Kenward-Roger  p-value adjustment: Tukey HSD | | | | | | | | | | | | | | |
| Gene | Sample sizes | | Contrast | | | | | Estimate | | df | | t-ratio | | p-value |
|  |  |  | Treatment 1 | | | Treatment 2 | |  |  |  |  |  |  |  |
| *Cactus* | n_HIGH/UNINOC_ =6 n_HIGH/VIRUS_ =8  n_LOW/UNINOC_ =7 n_LOW/VIRUS_ =10 | | HIGH/UNINOC | | | HIGH/VIRUS | | 0.57 | | 25.9 | | 0.48 | | 0.96 |
|  |  |  | HIGH/UNINOC | | | LOW/VIRUS | | -1.25 | | 27 | | -1.05 | | 0.72 |
|  |  |  | HIGH/UNINOC | | | LOW/UNINOC | | -1.04 | | 27 | | -0.81 | | 0.85 |
|  |  |  | HIGH/VIRUS | | | LOW/VIRUS | | -1.81 | | 25.5 | | -1.76 | | 0.31 |
|  |  |  | HIGH/VIRUS | | | LOW/UNINOC | | 1.61 | | 25.4 | | 1.43 | | 0.49 |
|  |  |  | LOW/VIRUS | | | LOW/UNINOC | | -0.21 | | 24.7 | | -0.19 | | 0.99 |
| *Dicer* | n_HIGH/UNINOC_ =7 n_HIGH/VIRUS_ =8  n_LOW/UNINOC_ =7 n_LOW/VIRUS_ =9 | | HIGH/UNINOC | | | HIGH/VIRUS | | 0.21 | | 24.7 | | 0.11 | | 0.99 |
|  |  |  | **HIGH/UNINOC** | | | **LOW/VIRUS** | | -8.17 | | 25.9 | | -4.13 | | **0.002** |
|  |  |  | HIGH/UNINOC | | | LOW/UNINOC | | -2.86 | | 25.6 | | -1.30 | | 0.57 |
|  |  |  | **HIGH/VIRUS** | | | **LOW/VIRUS** | | -8.92 | | 24.5 | | -4.73 | | **0.0004** |
|  |  |  | HIGH/VIRUS | | | LOW/UNINOC | | -3.08 | | 24.4 | | -1.54 | | 0.43 |
|  |  |  | **LOW/VIRUS** | | | **LOW/UNINOC** | | 5.84 | | 24.1 | | 3.04 | | **0.03** |
| *Hopscotch* | n_HIGH/UNINOC_ =7 n_HIGH/VIRUS_ =8  n_LOW/UNINOC_ =7 n_LOW/VIRUS_ =9 | | HIGH/UNINOC | | | HIGH/VIRUS | | -0.88 | | 24.6 | | -0.99 | | 0.75 |
|  |  |  | HIGH/UNINOC | | | LOW/VIRUS | | -1.26 | | 25.7 | | -1.38 | | 0.52 |
|  |  |  | HIGH/UNINOC | | | LOW/UNINOC | | -1.52 | | 25.4 | | -0.54 | | 0.95 |
|  |  |  | HIGH/VIRUS | | | LOW/VIRUS | | -0.38 | | 24.4 | | -0.46 | | 0.97 |
|  |  |  | HIGH/VIRUS | | | LOW/UNINOC | | -0.36 | | 24.3 | | -0.41 | | 0.98 |
|  |  |  | LOW/VIRUS | | | LOW/UNINOC | | -0.74 | | 24.1 | | -0.89 | | 0.81 |
| *Hymenoptaecin* | n_HIGH/UNINOC_ =7 n_HIGH/VIRUS_ =8  n_LOW/UNINOC_ =7 n_LOW/VIRUS_ =8 | | HIGH/UNINOC | | | HIGH/VIRUS | | 2.94 | | 24 | | 0.34 | | 0.99 |
|  |  |  | HIGH/UNINOC | | | LOW/VIRUS | | -7.04 | | 25 | | -0.77 | | 0.87 |
|  |  |  | HIGH/UNINOC | | | LOW/UNINOC | | 1.88 | | 25.1 | | 0.20 | | 0.99 |
|  |  |  | HIGH/VIRUS | | | LOW/VIRUS | | -9.99 | | 23.4 | | -1.20 | | 0.63 |
|  |  |  | HIGH/VIRUS | | | LOW/UNINOC | | -1.07 | | 23.5 | | -0.12 | | 0.99 |
|  |  |  | LOW/VIRUS | | | LOW/UNINOC | | 8.92 | | 23.1 | | 1.05 | | 0.72 |
| *Vitellogenin* | n_HIGH/UNINOC_ =6 n_HIGH/VIRUS_ =8  n_LOW/UNINOC_ =7 n_LOW/VIRUS_ =9 | | HIGH/UNINOC | | | HIGH/VIRUS | | 0.07 | | 25.8 | | 0.05 | | 0.99 |
|  |  |  | HIGH/UNINOC | | | LOW/VIRUS | | 2.12 | | 24 | | 1.26 | | 0.59 |
|  |  |  | HIGH/UNINOC | | | LOW/UNINOC | | 1.9 | | 24.9 | | 1.07 | | 0.71 |
|  |  |  | HIGH/VIRUS | | | LOW/VIRUS | | 2.05 | | 24.8 | | 1.46 | | 0.48 |
|  |  |  | HIGH/VIRUS | | | LOW/UNINOC | | 1.79 | | 24.5 | | 1.21 | | 0.63 |
|  |  |  | LOW/VIRUS | | | LOW/UNINOC | | -0.25 | | 23.5 | | -0.18 | | 0.99 |

**Supplemental Table 3:** Statistical analyses for Experiment 2. Significant results are in bold.

**Additional methods**

*Honey bee source*

For all experiments, honey bee subjects were derived from colonies managed at the Iowa State University Horticulture Research Station in Ames, IA. All were produced from open-mated queens from commercial stocks*.*

*Experiment 1: Short term larval starvation through restriction of alloparental care*

Five queens in five separate honey bee colonies were caged over a frame of drawn, empty comb, for 48h and allowed to lay eggs normally. After this interval, we removed the cages and placed the frames back into the colony; eggs were then allowed to hatch and larvae mature as normal. At 180h after the egg laying interval, a starvation or a control treatment was performed on each frame as follows. We removed the frames from the colony and brushed nurse bees off the frame completely. We then placed a wire push-in cage over half of the developing larvae, preventing nurse bees from accessing them for alloparental care and feeding; the other half of the larvae on the frame remained accessible to nurse bees. Each frame was outside of the colony <2 minutes during the treatment. We then returned each frame to the colony for 10 h, with bees maturing to approximately the developmental stage where larvae initiate spinning and stop feeding ^1^. After this point, we briefly removed the frames from the colonies and removed the push in cages, again allowing access by adult workers. Because honey bee larval development is highly regimented, focusing our restriction period to end at the spinning phase does not allow a window for compensatory feeding by the workers; i.e., they cannot feed the starved workers more after the treatment. After the restriction treatment, we returned the frames to the colony and the pupae were allowed to mature normally to the pharate stage (20 days after oviposition), after which we again removed the frames from the colony and placed them in an incubator at 34°C overnight. We placed separate wire cage enclosures over each treatment side (restricted vs normal) on each frame to keep emerging adults from the different groups separate. Once adults had emerged, we mixed the emerged bees from each treatment group, creating pools of bees for each treatment, i.e., NORM group and a RESTRICT group, each containing bees from five source colonies. Within the first 24h after emergence, they were then separated into observation cages. We repeated this full procedure twice, thus producing two separate generations of workers that experienced control or starvation conditions within the same colonies.

*Experiment 2: Long term colony-level diet quality manipulation*

To accomplish this, we constructed four experimental colonies as the mechanism for delivering the nutritional treatment to our focal larvae. Two colonies received high quality chestnut (*Castanea spp.*) pollen and the other two the lower quality rockrose (*Cistus* *sp.*) pollen. These pollens, both of which are naturally collected by honey bees, have been previously characterized for nutritional components and physiological effects on bees. *Cistus* pollen has approximately 50% the protein and amino acid content, and 25% antioxidant content, compared to *Castanea* pollen, but the two pollens have similar lipid and sugar content ^2^; *Cistus* pollen also contains lower levels of iron and calcium ^3^. When consumed by bees, these pollens produce divergent effects on gene expression, particularly genes associated with detoxification, immunity, and metabolism ^2–4^ and significantly affect how bees respond to disease ^2–4^and pesticide challenge ^5^. Therefore, hereafter these diet treatments are referred to as HIGH (the higher quality *Castanea sp.* pollen) and LOW (the lower quality *Cistus. sp. pollen*)*.* Each hive consisted of a standard single deep Langstroth frame hive body and contained ten frames, as follows: two drawn (i.e., covered in wax comb) but empty frames; two drawn frames with one side filled with honey; one frame with capped brood (pupae); 5 frames of foundation (to be removed later). We took great care to only use frames that contained no stored pollen.

We then created homogenous adult worker populations for each colony using methods modified from ^66^, which can be used to minimize between hive variation at the beginning of an experiment. To accomplish this, we brushed frames of nurse bees from four brood-containing frames from six different colonies (i.e., 24 frames of bees) into a single ventilated container. We mixed these gently to create a large homogenate of worker bees. From this mixture, approximately 4000 workers were measured out by volume (1.3 liters) and added to each of the four experimental hives. As such, each hive began the experiment with an approximately identical worker population from a single homogenized worker source. Each colony also received a standardized chemical queen signal, in the form of a commercially available ‘pseudoqueen’ (Mann Lake, LTD, Minnesota); use of this standardized signal removes variation in behavior that may occur due to different queen quality stimuli sensed by the workers.

To differentiate the hives by nutritional treatment, we fitted each with a restrictive pollen trap ^7^ that was constantly engaged. This device knocks the majority of pollen off of the legs of returning foragers, effectively precluding the colony from accessing pollen resources from the landscape while allowing for free flight of workers and collection of nectar. Instead, each colony received an experimental pollen treatment. 10 grams of pollen was placed into hives every other day by filling a small plastic dish ^8^ with *Cistus sp.* (HIGH) or *Castanea* (LOW) pollen, which was then pushed into the wax on a frame. Both pollen sources were purchased from Pollenergie® (France; ^2–4^). In every case, we replaced the pollen dish before it was completely consumed, ensuring each colony was provided *ad libitum* access to their respective pollen treatment while restricted from collecting other pollen from the outside environment.

Because the nutritional status of the workers decides the quality of food delivered to the larvae, we maintained these colonies under experimental conditions for multiple generations of workers to ensure the nutritional treatment was established and reduce any buffering the workers may have gained from previous experience. The first generation of workers were those in the original colony production, made up of the mixture of workers from wild type colonies; these would first start to be succeeded by the bees emerging from the capped brood frame initially present in the experimental colony. These bees, while experiencing normal larval development, emerged as adults into the nutritional treatment colonies. We also added a second and third frame of pupae to each colony once per week for the next three weeks. On the same interval, we also added frames of eggs to each colony; these provided larvae for the workers to care for to simulate a normal colony environment in the lead up to the production of the focal bees. We sourced these eggs from four different queens from our general apiary. Each time we placed a new frame into an experimental hive, a frame of undrawn foundation was removed to make space. Throughout this period, approximately 500 newly-emerged non-focal adults were added to each colony per week to ensure continuation of a stable population that experienced the hive nutritional treatment for their entire adulthood. At 21 days after the creation of the colony (the duration of a worker bees’ development from egg to adulthood), two frames, each partially filled with newly-laid eggs from different unmanipulated queens, were added to each colony. These eggs were allowed to develop normally within the colony until the they had reached the pharate stage (with cells capped), after which they were removed to a 34°C incubator for adult emergence. Unlike in Experiment 1, we kept the bees derived from each colony separated, accounting for colony source in our statistical models (see below). This procedure was repeated twice more, once 14 days after the original addition of focal eggs, and once more 14 days after that. Each iteration used eggs derived from different, randomly-selected queens. Thus, we produced three separate generations of adults that experienced each hive-level nutritional treatment.

*Body quality analysis of nutritional treatments* - *lipids*

Bees were processed for lipid quantification using a phospho‐vanillin spectrophotometric assay. Bees were placed in 5 ml of 2:1 chloroform:methanol, homogenized with a glass pestle and allowed to extract overnight. This extract was filtered through glass wool and adjusted to a constant volume. A subsample of 300 μl extract was dried, combined with 200 μl sulphuric acid, and then placed in a boiling water bath for 10 min. Then, 2 ml of the phospho‐vanillin reagent (6 mg vanillin per ml of water to 4 ml 85% phosphoric acid) was added. Samples were agitated and then removed from light to allow the reaction to occur for 15 min. Two hundred microlitres of each undiluted sample was pipetted into a 90‐well spectrophotometry plate, and absorbance at 525 nm was measured using a Spectra Max 190 multi‐well spectrophotometer. Absorbance measurements were converted to milligrams of lipid using a cholesterol standard curve.

*Cage Assays*

Acrylic cage dimensions: 10.6 × 10.16 × 7.62 cm.

Within 2h of the formation of each cage, a small weigh boat containing 600 µl of 30% sucrose solution was placed on the floor of each cage. Control cages received only sterile sucrose solution, i.e., they were not inoculated with IAPV (hereafter referred to as “UNINOC” treatment). Virus treatment cages received a 1:1000 dilution of a virus inoculum, identical to that described and used in ^3,9^ hereafter referred to as “VIRUS” treatment). After 16 h, the solution in each cage had been completely consumed by the workers; then, a top feeder of sterile 30% sucrose solution was added to the top of each cage, providing virus-free diet *ad libitum* for the remainder of the experiment. We monitored mortality each day for four (96h) days, the duration previously shown to be necessary to observe virus-induced mortality ^9,10^. At 36 hours post-infection (hpi), we sampled 3 live bees from each cage for virus titer and gene expression analysis.

In summary, for Experiment 1, there were four cage treatments: restricted+uninoculated (RESTRICT/UNINOC); restricted+virus inoculated (RESTRICT/VIRUS); normal+uninoculated (NORM/UNINOC); normal+virus inoculated (NORM/VIRUS). The first generation of workers reared under treatment conditions were used to produce 39 cages (n=9 for RESTRICT/VIRUS; n=10 for all others); the second generation produced 40 more cage (n=10 per treatment), for a final of 79 total cages (n=19 for RESTRICT/VIRUS; n=20 for all others). For Experiment 2, there were also four cage treatments (listed below), spread across three generations. Because there were more variable numbers of bees reared in the more natural but less controlled conditions, the number of cages produced from each generation was more variable, as follows. LOW/UNINOC (*Cistus* pollen + uninoculated; generation 1, n=8; generation 2, n=5; generation 3, n=8; total n=21); LOW/VIRUS ( *Cistus* pollen + IAPV inoculated; generation 1, n=9; generation 2, n=6; generation 3, n=7; total n=22 ); HIGH/UNINOC (*Castanea* pollen + uninoculated; generation 1, n=3; generation 2, n=6; generation 3, n=2; total n=11); HIGH/VIRUS (*Castanea* pollen + IAPV inoculated; generation 1, n=3; generation 2, n=6; generation 3, n=2; total n=11).

*Virus titration*

From the 6 bees collected from each cage at 36 hours post-treatment, we pooled bees by cage and extracted total body RNA from and measured IAPV titers from 10 randomly-selected cages from each treatment; this was done identically to the methods of Dolezal et al 2019a and Geffre et al. 2020. In short, RNA was extracted from each sample using Trizol reagent. This material was then cleaned and treated with DNAse to remove residual genomic DNA. RNA concentration was then equalized to 50ng/ul across all samples and 100 ng per reaction were measured via qPCR using the CFX384 TouchTM Real-Time PCR Detection System. Samples were run in duplicate, with cycle means used to calculate final values. Genome equivalents of IAPV were estimated for each sample via calculation against the RNA standard curve developed by ^9^.

*Gene expression*

Using the RNA extracted for virus titration (above), we measured gene expression via qPCR, as performed with the CFX384 TouchTM Real-Time PCR Detection System. Each sample was run in triplicate for each gene and we used the gene *actin* as an internal reference to normalize gene expression data, and used the 2^−ΔΔCT^ method ^12^ to calculate relative gene expression, with expression normalized to the internal control gene *actin*, and shown relative to the “UNINOC/NORM” treatment as the reference group in Experiment 1 and the “UNINOC/HIGH” treatment as the reference group in Experiment 2.

For each sample, we measured gene expression for five genes of interest, all of which play different important roles in immune response. Cactus plays a key role in the Toll immunity signaling pathway, with initiates production of antimicrobial peptides ^13^ ^14^. Hopscotch is a JAK tyrosine kinase ^15^ that is part of the JAK/STAT immune signaling pathway and previously has been associated with important honey bee immune responses ^16,17^. The antimicrobial peptide hymenoptaecin is involved in antibacterial and antiviral in honey bees ^18,19^. RNA viruses, including IAPV, are identified and destroyed by the RNA-interference pathway, including the enzyme dicer ^20^. Vitellogenin plays many roles, especially in honey bees; it acts as a storage protein, plays a regulatory role in honey bee behavior, and protects against oxidative and pathogen stress ^21^. By targeting genes that play a role in different facets of honey bee health and immune response, we can take our first steps in identifying how different types of developmental environments affect pathogen response in adults.

*References*

1. Jay, S. C. The development of honeybees in their cells. *J. Apic. Res.* (1963) doi:10.1080/00218839.1963.11100072.

2. Di Pasquale, G. *et al.* Influence of Pollen Nutrition on Honey Bee Health: Do Pollen Quality and Diversity Matter? *PLoS One* **8**, e72016 (2013).

3. Dolezal, A. G. *et al.* Interacting stressors matter: Diet quality and virus infection in honeybee health. *R. Soc. Open Sci.* (2019) doi:10.1098/rsos.181803.

4. Rutter, L. *et al.* Transcriptomic responses to diet quality and viral infection in Apis mellifera. *BMC Genomics* (2019) doi:10.1186/s12864-019-5767-1.

5. Leza, M., Watrous, K. M., Bratu, J. & Woodard, S. H. Effects of neonicotinoid insecticide exposure and monofloral diet on nest-founding bumblebee queens. *Proc. R. Soc. B Biol. Sci.* (2018) doi:10.1098/rspb.2018.0761.

6. Delaplane, K. S., Van Der Steen, J. & Guzman-Novoa, E. The COLOSS BEEBOOK, Volume I: standard methods for Apis mellifera research. *J. Apic. Res.* (2013).

7. DeGrandi-Hoffman, G. *et al.* Honey bee colonies provided with natural forage have lower pathogen loads and higher overwinter survival than those fed protein supplements. *Apidologie* (2016) doi:10.1007/s13592-015-0386-6.

8. Dolezal, A. G., Carrillo-Tripp, J., Miller, W. A., Bonning, B. C. & Toth, A. L. Pollen contaminated with field-relevant levels of cyhalothrin affects honey bee survival, nutritional physiology, and pollen consumption behavior. *J. Econ. Entomol.* (2016) doi:10.1093/jee/tov301.

9. Carrillo-Tripp, J. *et al.* In vivo and in vitro infection dynamics of honey bee viruses. *Sci. Rep.* (2016) doi:10.1038/srep22265.

10. Maori, E. *et al.* IAPV, a bee-affecting virus associated with colony collapse disorder can be silenced by dsRNA ingestion. *Insect Mol. Biol.* (2009) doi:10.1111/j.1365-2583.2009.00847.x.

11. Geffre, A. C. *et al.* Honey bee virus causes context-dependent changes in host social behavior. *Proc. Natl. Acad. Sci. U. S. A.* (2020) doi:10.1073/pnas.2002268117.

12. Livak, K. J. & Schmittgen, T. D. Analysis of relative gene expression data using real-time quantitative PCR and the 2-ΔΔCT method. *Methods* (2001) doi:10.1006/meth.2001.1262.

13. Valanne, S., Wang, J.-H. & Rämet, M. The Drosophila Toll Signaling Pathway . *J. Immunol.* (2011) doi:10.4049/jimmunol.1002302.

14. Richard, F. J., Holt, H. L. & Grozinger, C. M. Effects of immunostimulation on social behavior, chemical communication and genome-wide gene expression in honey bee workers (Apis mellifera). *BMC Genomics* (2012) doi:10.1186/1471-2164-13-558.

15. Binari, R. & Perrimon, N. Stripe-specific regulation of pair-rule genes by hopscotch, a putative Jak family tyrosine kinase in Drosophila. *Genes Dev.* (1994) doi:10.1101/gad.8.3.300.

16. Siede, R., Meixner, M. D. & Büchler, R. Comparison of transcriptional changes of immune genes to experimental challenge in the honey bee (Apis mellifera). *J. Apic. Res.* (2012) doi:10.3896/IBRA.1.51.4.05.

17. Evans, J. D. *et al.* Immune pathways and defence mechanisms in honey bees Apis mellifera. *Insect Mol. Biol.* (2006) doi:10.1111/j.1365-2583.2006.00682.x.

18. Casteels, P., Ampe, C., Jacobs, F. & Tempst, P. Functional and chemical characterization of hymenoptaecin, an antibacterial polypeptide that is infection-inducible in the honeybee (Apis mellifera). *J. Biol. Chem.* (1993).

19. Ryabov, E. V., Fannon, J. M., Moore, J. D., Wood, G. R. & Evans, D. J. The Iflaviruses Sacbrood virus and Deformed wing virus evoke different transcriptional responses in the honeybee which may facilitate their horizontal or vertical transmission. *PeerJ* (2016) doi:10.7717/peerj.1591.

20. Cerutti, H. & Casas-Mollano, J. A. On the origin and functions of RNA-mediated silencing: From protists to man. *Current Genetics* (2006) doi:10.1007/s00294-006-0078-x.

21. Harwood, G. P., Ihle, K. E., Salmela, H. & Amdam, G. V. Regulation of Honeybee Worker (Apis mellifera) Life Histories by Vitellogenin. in *Hormones, Brain and Behavior: Third Edition* (2017). doi:10.1016/B978-0-12-803592-4.00036-5.
